# ESPED survey on newly diagnosed immune thrombocytopenia in childhood: how much treatment do we give?

**DOI:** 10.1186/s40348-021-00121-z

**Published:** 2021-09-05

**Authors:** Hannah von Lukowicz, Paul-Gerhardt Schlegel, Christoph Härtel, Henner Morbach, Imme Haubitz, Verena Wiegering

**Affiliations:** Department of Pediatrics, University Hospital Würzburg, Josef-Schneider-Str. 7, 97080 Würzburg, Germany

**Keywords:** Pediatric immune thrombocytopenia, Acute ITP, Newly diagnosed ITP, ESPED, Autoimmunity, Treatment guidelines, Intravenous immunoglobulins, Bleeding score, Children

## Abstract

**Background:**

Immune thrombocytopenia (ITP) is an autoimmune disease associated with isolated thrombocytopenia, which is caused by an imbalance between platelet production and platelet destruction. Petechial and mucous membrane hemorrhages are characteristic of ITP, but life-threatening bleeding rarely occurs. Depending on the bleeding symptoms, ITP can be treated with glucocorticoids (GC), intravenous immunoglobulins (IVIG), or in severe cases, platelet transfusions. Mild bleeding does not necessarily require therapy. Using the German Surveillance Unit for rare Pediatric Diseases (ESPED) we conducted a prospective survey on ITP patients in all German Children's Hospitals between September 2018 and August 2019. We collected data on ITP, including the clinical course, therapy implementation recommendations (according to the Association of German Scientific Medical Societies guidelines), outcome, and influence of treatment regimens depending on the treating physician´s experience with ITP patients.

**Results:**

Of the 287 recorded cases of children with ITP, 268 questionnaires were sent to the authors. Two hundred seventeen of the questionnaires fulfilled the inclusion criteria. ITP affected boys and girls similarly, and the median age of manifestation was 3.5 years. The main reasons for hospitalization were thrombocytopenia, bleeding signs, hematomas, and/or petechiae. Bleeding scores were ≤ 3 in 96% of children, which corresponded to a low-to-moderately low risk of bleeding. No life-threatening bleeding was documented. The most common therapies were IVIG (*n* = 59), GC (*n* = 33), or a combination of these (*n* = 17). Blood products (i.e., red blood cells, platelet concentrate, and fresh frozen plasma) were given to 13 patients. Compared to the established guidelines, 67 patients were over-treated, and 2 patients were under-treated.

**Conclusions:**

Adherence to German ITP treatment guidelines is currently limited. To improve patient safety and medical care, better medical training and dissemination of the guidelines are required in line with targeted analyses of patients with serious bleeding events to identify potential risk constellations.

**Supplementary Information:**

The online version contains supplementary material available at 10.1186/s40348-021-00121-z.

## Background

Immune thrombocytopenia (ITP) is an autoimmune disease associated with isolated thrombocytopenia (platelet count < 100 × 10^9^/L), caused by an imbalance between platelet production and platelet destruction [1]. Evidence of specific autoantibodies can ensure an ITP diagnosis, but in the absence of evidence, the ITP diagnosis is based on exclusion. With an incidence of 3–5/100,000 individuals, ITP is the most common cause of thrombocytopenia in children [2]. Petechial hemorrhage and mucous membrane hemorrhage are characteristic of ITP, but life-threatening bleedings, including gastrointestinal and intracerebral bleeding, rarely occur. In most cases, the disease is self-limiting in children and does not require specific therapy [3]. For severe courses of ITP, the available treatments are intravenous immunoglobulins (IVIG), glucocorticoids (GC), and platelet substitution. Guidelines for the diagnosis and treatment of ITP were established in 2010 by the Association of Scientific Medical Societies in Germany (AWMF guidelines; updated in 2018) [4]. These guidelines recommend a watch-and-wait strategy for cases of mild and moderate bleeding without significant bleeding signs; the use of GC or IVIG for cases of relevant mucosal bleeding; and the use of platelet concentrates in cases of life-threatening bleeding [4].

The present study aimed to collect objective data on current treatment decisions and approaches for patients with ITP in Germany. We conducted a prospective German Surveillance Unit for rare Pediatric Diseases (ESPED) survey at German children’s hospitals to collect up-to-date data about the reason for admission, clinical course, therapy implementation recommendations (based on AWMF guidelines), outcome, and therapy differences depending on the treating physician’s experience with ITP patients. These data might provide a benchmark for developing strategies to improve patient care in the future.

## Methods

Between 1 September 2018 and 31 August 2019, all pediatric hospitals nationwide were contacted monthly by the ESPED to notify and remind the staff that all hospitalized patients newly diagnosed with ITP should be recorded by completing a questionnaire (see Supplementary material) [5]. The questionnaires were checked by the ESPED, which was the central receiving point. Then, the data were anonymized and sent to the authors of the present study. Data were entered into an Excel table for further statistical evaluations.

Thrombocytopenia due to other causes (e.g., neonatal, hereditary, oncological, or an acute severe infection) were excluded, based on information given in the questionnaire (i.e., blood count, pre-existing conditions, medication, and family history).

### Statistics

Descriptive statistics were performed with IBM SPSS Statistics 24. All other statistical analyses were performed with MEDAS software (Grund EDV, Margetshöchheim). Categorical variables were compared between two groups, either using the chi square test or, when the values were expected to be small, with the exact test, according to Fisher, and the exact test, according to Mehta and Patel. Continuous measurements were compared between two groups with the Mann-Whitney *U* test. Comparisons of more than two groups were performed with the rank variance analysis, according to Kruskal and Wallis. Kendall's ranking correlation was used to describe the relationship between age and platelets. Correlation between severe bleeding and platelet levels were calculated with Pearson’s rank correlation coefficient. In addition, relative risk curves were calculated with the log-rank test. *P* values < 0.05 were considered significant.

The bleeding score mentioned in this study was determined retrospectively, based on the bleeding signs given in the questionnaire. The modified Buchanan bleeding Score [6] provides an overall grade from 0 to 5 which incorporates skin, oral, and mucosal bleeding. Grade 0–2 includes bleeding of the skin only, grade 3 includes mucosal bleeding, and grade 4–5 is any bleeding that requires immediate medical attention or is life threatening (Table 1).

**Table 1:** Modified Buchanan bleeding score [6]

Grade		
0	None	No new hemorrhage of any kind
1	Minor	Few petechiae (≤ 100 total) and/or ≤ 5 small bruises (≤ 3 cm diameter), no mucosal bleeding
2	Mild	Many petechiae (> 100 total) and/or > 5 large bruises (> 3 cm diameter)
3	Moderate Low risk	Blood crusting in nares, painless oral purpura, oral/palatal petechiae, buccal purpura along molars only, mild epistaxis ≤ 5 min
	Moderate High risk	Epistaxis > 5 min, hematuria, hematochezia, painful oral purpura, significant menorrhagia
4	Severe	Mucosal bleeding or suspected internal hemorrhage (brain, lung, muscle, joint etc.) that requires immediate medical attention or intervention
5	Life threatening/fatal	Documented intracranial hemorrhage or life threatening or fatal hemorrhage at any site

To investigate adherence to guidelines, the therapy and the bleeding score determined from the questionnaire were compared to the therapy recommended by the current guidelines. The results were expressed by defining a new variable, called *guideline adherence*, with three categories: over-treatment, under-treatment, and guideline-compliant treatment.

## Results

### Response rate, exclusion criteria, and evaluated cases

From the 287 recorded cases with ITP during the study period, we received 268 questionnaires (return rate 93%). We excluded 51 questionnaires, due to insufficient data quality (i.e., false notification/double notification). Thus, 217 questionnaires were analyzed. Patients < 18 years of age with newly diagnosed ITP were included. Exclusion criteria were the presence of a thrombocytopenia caused by other entities, including neonatal (< 4 weeks old), hereditary, rheumatic or systemic (auto)immune diseases or malignant disease, or related to a severe infection, and the presence of chronic ITP. This ESPED survey did not provide a standardized follow-up.

### Clinical characteristics

#### Age and gender distributions

The median age was 3.54 years (range 81 days to 17.96 years). There was no gender disparity, with 105 boys (48.4%) and 112 girls (51.6%). Age and gender of affected children had no significant influence on the bleeding score, the therapy, or guideline adherence. However, platelet values were positively correlated with age: the older the patient, the higher the platelet count at admission (tau = 0.09, *p* = 0.043), while platelet values and gender were not correlated.

#### Seasonal influences

There was no seasonal influence on the incidence of ITP, based on a comparison of the number of patients admitted with ITP in autumn/winter (October–March; *n* = 122; 56.2%) vs. spring/summer (April–September; *n* = 95; 43.8%).

#### Preceding infections, medications, and immunizations

Of the 217 patients included, 142 (65.4%) had a preceding infection. The average onset of the infection was 11.69 days (± 10.76 days) before hospitalization. Prior to admission, 61 children (33.9%) had received drug therapy, and of these, 21 had received a chronic medication (vitamin D, macrogol, inhaled GC, etc.), 29 had received an acute medication (antibiotics, inhaled salbutamol, and others), 9 had received ibuprofen, and 2 had received tranexamic acid. In the 30 days prior to admission, 8 children had received immunizations, including vaccines against measles, mumps, and rubella (*n* = 2), measles, mumps, rubella, and varicella (*n* = 4), and meningococcal group C (*n* = 1), and meningococcal group B (*n* = 1) infections, respectively.

### Reasons for admission, platelet counts, and bleeding severity

In most cases, the reasons for admission were thrombocytopenia (*n* = 86, 39.6%), bleeding (*n* = 114, 52.5%), and hematoma (*n* = 41, 18.9%) or petechiae (*n* = 56; 25.8%). Questionnaire respondents had the option to give multiple answers to this question.

The frequency distribution of the different ITP severities is shown in Table 2, based on the bleeding score by the modified Buchanan bleeding score [6, 7], and the platelet count. In this study we had only one patient with a bleeding score of 3b, so that we merged score 3a and 3b in this table. Platelet levels > 20 × 10^9^/L were observed in 2/3 of patients with a bleeding score of 0. In contrast, all patients with a bleeding score > 3 had platelet levels < 20 × 10^9^/L. 84% of patients with bleeding score ≤ 2 (*p* = 0.05) had platelet levels < 20 × 10^9^/L.

**Table 2:** Frequency distribution of ITP severities and bleeding score

	Total	Platelet count × 10^9^/l				Bleeding score adapted to Buchanan et al.					
	*N* (%)	0–9	10–19	20–29	> 30	0 (None)	1 (Minor)	2 (Mild)	3 (Moderate)	4 (Severe)	5 (Life threatening)
No therapy	95 (44%)	49	19	16	11	4	31	36	23	1	0
IVIG	59 (27%)	51	4	3	1	3	13	18	24	1	0
GC	33 (15%)	22	9	1	1	1	4	8	19	1	0
IVIG und GC	17 (35%)	15	2	0	0	0	3	4	9	1	0
Blood products (RBC, PC, FFP)	1 (1%)	0	1	0	0	0	0	0	0	1	0
Blood products and IVIG/GC	12 (6%)	12	0	0	0	0	1	1	7	3	0
Total	217 (100%)	149 (69%)	35 (16%)	20 (9%)	13 (6%)	8 (4%)	52 (24%)	67 (31%)	82 (38%)	8 (4%)	0 (0%)
Mean platelet count (/μl)	6000					23500	11923	10388	7390	7375	-

*RBC* red blood cells, *PC* platelet concentrate, *FFP* fresh frozen plasma

The relationship between platelet levels at hospitalization and the bleeding score was significant (*r* = − 0.230, *p* = 0.0018).

During hospitalization, 9 children (4.4%) developed severe bleeding. In these 9 cases, no life threating bleeding or intracranial bleeding are reported. This complication was not significantly correlated with the platelet levels at admission (Pearson *r* = − 0.050, *p* = 0.478) nor with the minimum platelet levels observed during the inpatient stay (Pearson *r* = − 0.102, *p* = 0.153).

### Course of platelet counts

During the inpatient stay, the platelet counts declined further in 34% (*n* = 74) of the patients. Thirty-eight patients (18%) never reached values above 20 × 10^9^/L, and 66 patients (30%) never reached values above 30 × 10^9^/L. On average, the patients were in the hospital for 6 ± 4 days. The minimum platelet values occurred at a mean of 1.5 ± 1.1 days after admission. Platelet levels reached 20 × 10^9^/L at a mean of 5.5 ± 6 days after admission and 30 × 10^9^/L at a mean of 6.5 ± 7 days after admission. We found a significant correlation between the minimum platelet count and the severity of the bleeding score (*p* < 0.001) and the minimum platelet count and the length of the hospital stay (*p* < 0.001). At discharge, platelet counts were significantly higher in children that had received therapy (88 ± 72 × 10^9^/L) than in children that had not received therapy (36 ± 65 × 10^9^/L; *p* < 0.001). However, when the platelet counts were evaluated on an outpatient basis after discharge there was no longer a significant difference in platelet counts between groups stratified to therapy.

### Diagnostics

The most common initial supplemental diagnostics were a manual differential blood count (*n* = 154; 71.2%) and abdominal ultrasound (*n* = 124; 57.1%). A bone marrow aspiration was performed in 12 patients (5.6%), and screening for human platelet antibodies was performed in 19 patients (8.7%). Rarely, *Helicobacter pylori*-diagnostics (1.8%), immunodeficiency diagnostics (2.8%), and von Willebrand factor, type 2B diagnostics (5.5%) were performed.

### Therapy

Overall, 95 (43.8%) patients with ITP were not treated during the inpatient stay (Table 2). Their platelet counts ad admission were significantly higher (15 ± 15.7 × 10^9^/L) than the counts in children that were treated (6 ± 5.8 × 10^9^/L; *p* < 0.0001). The most common therapy was IVIG alone (*n* = 59), followed by GC (*n* = 33), and the combination of IVIG and GC (*n* = 17). Some patients required blood products, including red blood cells (*n* = 2), platelet concentrate (*n* = 11), and fresh frozen plasma (*n* = 1). Four patients received tranexamic acid as a co-medication. The most used GC was prednisolone (2–4 mg/kg)/day. A low platelet count was significantly correlated with the use of IVIG (*p* < 0.0001) and GC (*p* = 0.015). In addition, a severity of bleeding was significantly correlated with the use of GC and blood products (*p* < 0.0006).

### Side effects

Acute documented side effects due to therapy occurred in 14 of 88 patients that received IVIG therapy (15.9%). The most frequent symptoms were headaches, nausea, vomiting, and fever. Side effects due to other medications were not reported. Moreover, other longer-term side effects were not detected.

### Influence of pediatric hospital size and equipment

We compared patient characteristics and therapy regimens between hospitals with different annual numbers of ITP cases (self-estimated case counts), with/without intensive care beds, and with/without a hematological outpatient clinic Table 3. We found that 58% of patients were treated in hospitals with a case count of 0–5 patients/year, 27% were treated in hospitals with a case count of 6–10 patients/year, and 25% were treated in hospitals with a case count of > 10 patients/year. We found that higher numbers of ITP cases/year were associated with lower platelet counts at admission (*p* = 0.0052). In addition, patients who visited hospitals with a hematological outpatient clinic had significantly lower minimal platelet counts (*p* = 0.03) and were more likely to receive IVIG (*p* = 0.001) than patients without access to a hematological outpatient clinic.

**Table 3:** Therapy regime depending on the number of patients treated per year

	Number of patients treated per year						
			**0–5**		**6–10**		**> 10**
		*n*	%	*n*	%	*n*	%
Distribution		116	58	54	27	29	15
Platelet count/μl		8444 ± 9115		13926 ± 15410		6776 ± 12310	
Intensive care unit	**No**	36	31.3%	0	0.0%	0	0.0%
	**Yes**	79	68.7%	54	100%	29	100%
Hematological outpatient clinic	**No**	94	81.0%	34	62.9%	3	10.3%
	**Yes**	22	18.9%	20	37.0%	26	89.7%
Therapy	**No**	50	43.1%	25	46.3%	9	31.0%
	**Yes**	66	56.9%	29	53.7%	20	68.9%
Therapy modality	**None**	50	43.1%	25	46.3%	9	31.0%
	**IVIG**	27	23.3%	18	33.3%	13	44.8%
	**GC**	22	18.9%	6	11.1%	2	6.9%
	**IGIV and GC**	10	8.6%	2	3.7%	4	13.8%
	**Blood products alone**	0	0.0%	0	0.0%	1	3.5%
	**Blood products, IVIG + GC**	7	6.0%	3	5.56%	0	0.0%

### Guideline adherence

In our cohort, 148 patients (68%) received treatment adherent to the current guideline, 2 patients (1%) were under-treated, and 67 patients (31%) were over-treated. Guideline adherence was not significantly correlated with the availability of an intensive care unit, the presence of a hematological outpatient clinic, or the number of ITP patients treated/year. The cohort of patients with over-treatment (*p* < 0.001) included all 13 patients that received blood products, 34 patients that received IVIG, 13 that received GC, and 7 that received a combination of IVIG and GC.

Patients that were over-treated had significantly fewer platelets at admission than those that received guideline-compliant treatment (5.9 ± 4.7 × 10^9^/L vs. 12 ± 14 × 10^9^/L, *p* = 0.0036). In comparing the different forms of therapy, we found that platelet levels were lowest in patients treated with a combination of blood products and either GC therapy or IVIG (3.8 ± 1.8 × 10^9^/L). Platelet levels improved with the combination of IVIG and GC (4 ± 4.5 × 10^9^/L) and with monotherapy of IVIG (6 ± 4.6 × 10^9^/L). The highest platelet levels were observed in patients treated with GC (7.5 ± 8.5 × 10^9^/L).

In comparing patients with different bleeding scores, over-treatment was significantly more common among patients with no or mild bleeding propensity (*p* < 0.001) than in patients with higher bleeding scores.

In a multivariate logistic regression analysis, we investigated whether the guideline-compliant treatment was influenced by children’s age, gender, bleeding score, platelet count, or reason for admission. We found that the bleeding score (odds ratio [OR] 2.82; 95% confidence interval [95%CI] 1.88–4.24), the platelet count (OR 1.13; 95%CI 1.06–1.20), and ‘bleeding’ as the reason for admission (OR 0.38; 95%CI 0.19–0.77) had a significant impact on medical decisions. The probability of guideline-compliant treatment nearly tripled at each “increase in bleeding score.”

## Discussion

Newly diagnosed ITP is the most common cause of childhood thrombocytopenia [2]. Its etiopathogenesis remains largely unclear and, in the absence of autoantibody detection, the diagnosis is determined by exclusion.

Our current ESPED cohort appeared to be representative of the general ITP population, in terms of age and gender distribution and the maximum frequency of ITP episodes in infancy [8]. We found no seasonal influence on the incidence in this cohort. However, viral infections are likely to be associated with newly diagnosed ITP; thus, the previously described dependencies may have been more related to viral outbreaks than to the season per se [9]. Appropriately, the data presented here reflected the expected associations between previous infections and immunizations and the occurrence of newly diagnosed ITP.

ITP is characterized by petechial skin and mucosal hemorrhages, but in severe cases of thrombocytopenia, life-threatening bleeding has been described, particularly when it involves gastrointestinal and intracerebral bleeding. The actual incidence of serious spontaneous bleeding is unclear, but it is certainly low (< 5%) [10]. Previous studies have not shown a linear correlation between bleeding severity and the absolute platelet count, but severe bleeding events were always associated with < 20 × 10^9^/L platelets [9, 11, 12]. We showed that the bleeding score was correlated with platelet count; nevertheless, a low platelet count did not necessarily translate into a high risk of bleeding. In this prospective ESPED survey, no life-threatening hemorrhages occurred.

There is an ongoing discussion among experts regarding therapy indications. The recommended therapy guidelines differ greatly among the USA, UK, and Germany [13–17]. In the 1990s, the ITP treatment rate was comparatively high in Germany (40% vs. 14% in the UK), particularly treatment with IVIG (60% vs. 23%, [11]). However, despite the different therapy regimens, comparable outcomes were observed for patients with serious bleeding. Currently, a discordance in therapy indications continues among different countries, and the alleged over-treatment rate has continued, despite revised guidelines. In the USA, > 90% of patients are treated, although just under 15% actually show bleeding symptoms [15]; the UK has a more restrictive approach where approximately 16% of patients are treated [18].

Our present ESPED survey on ITP in children and adolescents showed that patients with ITP were being over-treated, and the rate of guideline adherence was low. The significant correlation we observed between the platelet count at admission and therapy initiation suggested that therapy decisions were probably based on the platelet count, which is not recommended in current guidelines.

Compared to a previous ESPED survey (Table 4) [11], our survey showed that the watch-and-wait strategy is now more frequently followed in Germany (44% vs. 14%), and the use of IVIG has decreased (35% vs. 61%). Nevertheless, decisions about therapy indications continue to focus on the platelet count, rather than on the individual bleeding score, as recommended in the guidelines. All our included patients in this study were hospitalized and there might be a great proportion of patients in addition receiving therapy in the ambulatory setting. In context of a significant amount of patients with mild bleeding symptoms in the hospitalized setting, not only the indication for therapy, but also the hospitalization rate seems to be (too) high.

**Table 4:** Modified Buchanan bleeding score [6]

Grade		
0	None	No new hemorrhage of any kind
1	Minor	Few petechiae (≤ 100 total) and/or ≤ 5 small bruises (≤ 3 cm diameter), no mucosal bleeding
2	Mild	Many petechiae (> 100 total) and/or > 5 large bruises (> 3 cm diameter)
3	Moderate Low risk	Blood crusting in nares, painless oral purpura, oral/palatal petechiae, buccal purpura along molars only, mild epistaxis ≤ 5 min
	Moderate High risk	Epistaxis > 5 min, hematuria, hematochezia, painful oral purpura, significant menorrhagia
4	Severe	Mucosal bleeding or suspected internal hemorrhage (brain, lung, muscle, joint etc.) that requires immediate medical attention or intervention
5	Life threatening/fatal	Documented intracranial hemorrhage or life threatening or fatal hemorrhage at any site

To our knowledge, most studies have shown no connection between the initial therapy for ITP and the long-term outcomes; i.e., severe bleeding events cannot be safely prevented with any therapy [11]. However, there is some evidence that the long-term outcome is not influenced by the initial therapy [19].

Treatment effect profiles and response rates have indicated that the short-term response to IVIG appears to occur slightly earlier than other therapies. On the other hand, GC is easily applicable in an outpatient setting, it is significantly less expensive, and it had comparable adverse reaction rates, compared to other therapies [2, 9].

In the context of over-treatment for ITP, the attending physician must be clearly aware of the side effect profiles of different treatments and consider the probability of a low spontaneous bleeding rate. Thus, it is necessary to perform a critical benefit-risk analysis, together with the legal guardians, before starting treatment. In particular, the use of blood products (platelet concentrates and IVIG) in patients with mild bleeding signs appeared to be disproportionately high. Medical training and stewardship programs may help to restrict these potentially harmful treatments.

The rarity of serious bleeding events makes it unlikely that future randomized studies on therapy will be possible. Consequently, in the risk-benefit assessment, a grey zone will persist between observations (watch-and-wait, in hospital) and the indication for therapy [16]. Patients with low platelet counts and few bleeding signs continue to be treated much more frequently than recommended. Unlike cytopenia being linked to cell production disorders, like leukemia or (chemo-)therapy-associated cytopenia, in ITP, bleeding events can occur at different rates in patients with the same platelet counts. This finding was consistent with the relatively short in vitro bleeding times reported previously in studies on patients with ITP [20]. Moreover, this lack of correlation may explain the propensity of over-treatment, because experiences from other blood diseases (e.g., from hemato-oncology) are not generally applicable to ITP therapy.

## Limitations

The main limitation of this study was that the patient cohort did not include children or adolescents with severe bleeding (bleeding score 5) and only one patient with bleeding score 3b. Thus, no conclusions or therapy recommendations could be derived for this group of patients. Furthermore, only cases with hospital admission are included in this study (due to ESPED reporting systems) and outpatient courses are not systematically reported. Patients without hospital admission could not be included in this study.

Furthermore, we used the modified bleeding score for our calculations, which has so far only been used in a small cohort for therapy decisions [6]. Despite that the AWMF guidelines suggest using the modified Buchanan score, we therefore decided to use it in our study. However, a prospective evaluation of this score would be helpful to develop an accepted standard for bleeding scores. Finally, we missed to include the score in our questionnaire so that we had to calculate it retrospectively.

## Conclusions

The current ESPED survey showed that clinicians have not been treating ITP with sufficient adherence to guidelines. Based on this survey, we confirmed the low incidence of spontaneous bleeding events (particularly life-threatening bleeding) among patients with ITP. Therefore, a restrained, symptom-oriented therapy seems justified. Better medical training on current therapy guidelines is urgently needed to avoid over-treatment, to improve assessments of individual risk, including side effects, and to improve patient safety and care. In the future, an accurate analysis of patients with ITP that are known to experience serious bleeding events might identify potential risk constellations. That information could perhaps increase acceptance of the watch-and-wait approach in the future.

## Supplementary Information

**Additional file 1.** ESPED questionnaire.

## Data Availability

The datasets used and/or analyses performed during the current study are available from the corresponding author upon reasonable request.

## Abbreviations

ITP: Immune thrombocytopenia
ESPED: Erhebungseinheit für seltene pädiatrische Erkrankungen in Deutschland (translation: German Surveillance Unit for rare Pediatric Diseases)
AWMF: Association of Scientific Medical Societies in Germany
OR: Odds ratio
IVIG: Intravenous immunoglobulins
GC: Glucocorticoids
