# Supplementary material for: ESPED survey on newly diagnosed immune thrombocytopenia in childhood: how much treatment do we give?
Source: Mol Cell Pediatr. 2021 Sep 5;8:11. doi: 10.1186/s40348-021-00121-z (PMC8419130; doi:10.1186/s40348-021-00121-z)
Supplement: Supplementary file 1 — Additional file 1. ESPED questionnaire. [file 40348_2021_121_MOESM1_ESM.docx]

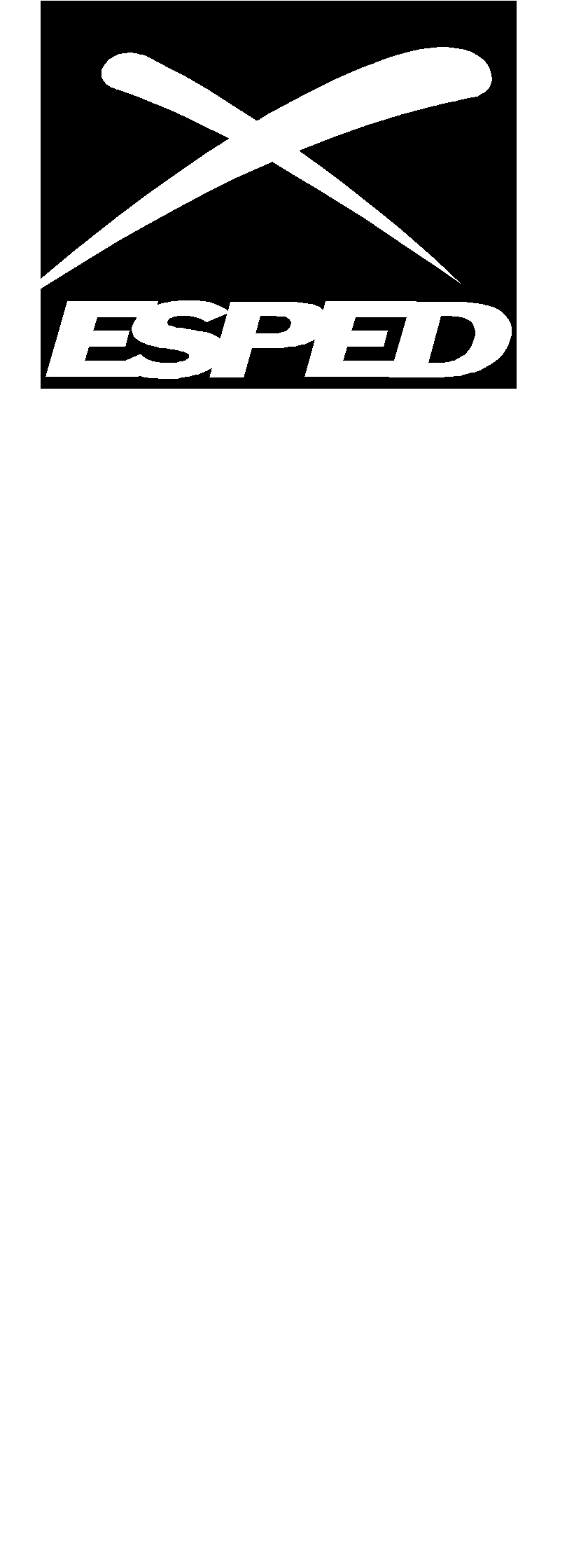

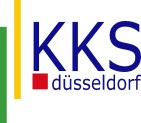
Erhebungseinheit für seltene pädiatrische Erkrankungen

in Deutschland

Forschungsstelle für pädiatrische Epidemiologie bei der Deutschen Gesellschaft für Kinder- und Jugendmedizin e.V.

Akute Immunthrombozytopenie (ITP) im Kindes- und Jugendalter

|  | ESPED  Arbeitsgruppe am KKS der  Heinrich-Heine-Universität Düsseldorf  z.Hd. Frau Heinrich  Postfach 10 22 44  40013 Düsseldorf | ⇦ | ***Rücksendung an nebenstehende Adresse erbeten!*** | |
| --- | --- | --- | --- | --- |
|  |  |  | IDNO:  LNR:  Meldemonat:  ESPED-Eingang: | *«IDNO»*  **«LNR»**  «MONAT» |

Berichtende Kinderklinik: Ansprechpartner für Rückfragen (vollständiger Name/Stempel):

*«KLINIK»* *..........................................................................................................................*

*«ABT»*

*«STR»* Tel.: ..................................................................................................................

*«PLZ» «ORT»*

Fax: .................................................……………...............................................

Email: ..................................................……………...........................................

#### Patienten-Datenblatt (verbleibt in der ESPED-Geschäftsstelle!)

Geburtsdatum (Tag/Monat/Jahr): I____ I____I_________I

Geschlecht: □ weiblich

□ männlich

Postleitzahl Wohnort: I__I__I__I__I__I

Stationäre Aufnahme (Tag/Monat/Jahr): I____I____I_________I . . . . . . . . . . .

Entlassungsdatum (Tag/Monat/Jahr): I____I____I_________I . . . . . . . . . . .



Akute Immunthrombozytopenie (ITP) im Kindes- und Jugendalter

- Klinik-Fragebogen –

Allgemeingültig beim Ausfüllen:

- Nichtzutreffendes bitte streichen (z.B. bei Maßeinheiten-Angaben)
- Mehrfachnennungen sind immer möglich
- Bitte kennzeichnen Sie nicht zu beantwortende Fragen und fahren mit der nächsten Frage fort.
- Bei Messwerten nennen Sie bitte das zum gefragten Zeitpunkt am Weitesten von der Norm abweichende, aber plausible Ergebnis.

#### 1. Patientendaten

***Wird von ESPED ausgefüllt!***

Geburtsdatum: I_____I_________I PLZ Wohnort: _______XX Alter bei Aufnahme: _______ Tage

Geschlecht: weiblich / männlich Alter bei Entlassung: _______ Tage

**2. Anamnestische Daten**

Vorausgegangener Infekt? □ nein □ ja Wenn ***ja***, Beginn. . . . . . . . . Tage vor Aufnahme,

Ende . . . . . . . . .Tage vor Aufnahme

In den letzten 4 Wochen vor Erkrankungen vorausgegangene Lebendimpfung?

□ nein □ ja Wenn ***ja***, welche: . . . . . . . . . . . . . . . . . . . . . . . . . . . . . . . . . .

Wenn ***ja***, an welchen Tagen vor Aufnahme: . . . . . . . . . . . . .

Unauffällige Thrombozytenwerte in Vorgeschichte dokumentiert? □ nein □ ja

□ unbekannt

Medikamente vor Aufnahme (<3 Wochen): □ nein □ ja Wenn ***ja***, welche: . . . . . . . . . . . . . . . . . . . . . . . . . . . . . . . . . .

. . . . . . . . . . . . . . . . . . . . . . . . . . . . . . . . . . . . . . . . . . . . . . . .

Vorerkrankungen? □ nein □ ja Wenn ***ja****, welche:* . . . . . . . . . . . . . . . . . . . . . . . . . . . . . . . . . . . . . . . . . . . .

□ unbekannt . . . . . . . . . . . . . . . . . . . . . . . . . . . . . . . . . . . . . . . . . . . . . . . . . . . . . . . . . .

Aufnahmegrund: □ auffälliger Laborwert □ Intervention □ Blutung

□ anderes: . . . . . . . . . . . . . . . . . . . . . . . . . . . . . . . . . . . . . . . . . . . . . . . . . . . . . . . . . . . . . . . . . . . . . .

Familienanamnese: □ unauffällig □ Thrombozytopenie □ Hämatologische Auffälligkeiten

2/3

**3. Blutung zum Zeitpunkt der stationären Aufnahme**

□ Keine Blutung

□ Leicht: 🢚 □ Petechien □ Hämatome □ leicht stillbares Schleimhautbluten □ Mikrohämaturie

□ Schwer: 🢚 □ Tamponade erforderlich □ Massiver Blutverlust (Transfusion erforderlich) □ Menorrhagie

□ Lebensbedrohlich: 🢚 □ ZNS Blutung □ Todesfall (Bitte anonymisierten Arztbrief zusenden)

Sind schwere Blutungssymptome während des Krankenhausaufenthaltes aufgetreten? □ nein □ ja

Wenn ***ja***, am wievielten Tag des Krankenhausaufenthalts? . . . . . . Tag

**4. Diagnostik**

**Thrombozytenzahl:**

Zählmethode: □ Mikroskop □ Zähl-Automat □ EDTA □ Citrat

Wert am Aufnahmetag: . . . . . . . . . . . . . . . . . /µl

Verlauf:

Niedrigster Wert: . . . . . . . . . . . . . . . . . /µl am wievielten Krankenhaustag: . . . . . . .

Wert > 20.000/µl am wievielten Krankenhaustag: . . . . . . .

Wert > 30.000/µl am wievielten Krankenhaustag: . . . . . . .

Hb-Wert bei Aufnahme: . . . . . . . . . . . . . . . . . g/dl

Leukozyten bei Aufnahme: . . . . . . . . . . . . . . . . . µl

Immature Plättchenfraktion: . . . . . . . . . . . . . . . . . %

Mittleres Plättchenvolumen: . . . . . . . . . . . . . . . . . fl

**Weitere Diagnostik:**

Antithrombozytäre Antikörper: □ nicht untersucht □ nicht vorhanden □ ja

Knochenmarkpunktion: □ nein □ ja Wenn ***ja****,* Ergebnis: . . . . . . . . . . . . . . . . . . . . . . . . . . . . . . . . . . . . . . . .

Sonographie Abdomen: □ nein □ ja Wenn ***ja****,* Ergebnis: . . . . . . . . . . . . . . . . . . . . . . . . . . . . . . . . . . . . . . . .

Manuelles Differentialblutbild: □ nein □ ja Wenn ***ja****,* Ergebnis: . . . . . . . . . . . . . . . . . . . . . . . . . . . . . . . . . . . . . . . .

Virusdiagnostik (insb. CMV, HCV, HIV, EBV, VZV): □ nicht untersucht □ negativ □ positiv

Wenn ***positiv***: Nachweis von: . . . . . . . . . . . . . . . . . . . . . . . Therapie: . . . . . . . . . . . . . . . . . . . . . . . . . . . . . . . . . . .

Nachweis von: . . . . . . . . . . . . . . . . . . . . . . . Therapie: . . . . . . . . . . . . . . . . . . . . . . . . . . . . . . . . . . .

Nachweis von: . . . . . . . . . . . . . . . . . . . . . . . Therapie: . . . . . . . . . . . . . . . . . . . . . . . . . . . . . . . . . . .

Helicobacter pylori Diagnostik: □ nicht untersucht □ negativ □ positiv, Therapie: . . . . . . . . . . . . . . . . . . . . . . . . . . .

. . . . . . . . . . . . . . . . . . . . . . . . . . . . . . . . . . . . . . . . . . .

Immundefektdiagnostik: □ nicht untersucht □ negativ □ positiv, Ergebnis: . . . . . . . . . . . . . . . . . . . . . . . . . . . . . .

vWF Typ 2B: □ nicht untersucht □ negativ □ positiv

**5. Therapie**

□ keine Medikamente □ keine Blutprodukte

| Medikamente/ Blutprodukte | Indikation | | | Beginn  am wievielten  Krankenhaustag? | Präparat:  Dosis/Tag | Dauer  [Tage] |
| --- | --- | --- | --- | --- | --- | --- |
|  | Plättchenzahl | Blutungssymptome | Andere |  |  |  |
| Immunglobuline (g) |  |  |  |  |  |  |
| Corticosteroide (mg) |  |  |  |  |  |  |
| Erythrozytenkonzentrat |  |  |  |  |  |  |
| Thrombozytenkonzentrat |  |  |  |  |  |  |
| nPlate (µg) |  |  |  |  |  |  |
| Revolade (mg) |  |  |  |  |  |  |
| andere |  |  |  |  |  |  |

Nebenwirkungen im unmittelbaren zeitlichen Zusammenhang mit Medikamenten? □ nein □ ja

3/3

Wenn ***ja***: Wie viele Stunden nach Medikamentengabe? . . . . . . . . . h

Welche Medikamente? . . . . . . . . . . . . . . . . . . . . . . . . . . . . . . . . . . . . . . . . . . . . . . . . . . . . . . . . . . . . . . . . . . . . .

Symptome: □ Kopfschmerzen □ Nausea □ Erbrechen □ Fieber

□ andere: . . . . . . . . . . . . . . . . . . . . . . . . . . . . . . . . . . . . . . . . . . . . . . . . . . . . . . . . . . . . . . . . . . . .

**6. Komplikationen, Outcome**

Komplikationen? □ nein □ ja Wenn ***ja***, welche . . . . . . . . . . . . . . . . . . . . . . . . . . . . . . . . . . . . . . . . . . . . . .

Ambulante Weiterbehandlung in der Klinik? □ nein □ ja

Kontrolle des Blutbildes? □ nein □ ja Wenn ***ja***, wie oft? . . . . . . . . . . . . . . . . . . . . . . . . . . . . . . . . . . . . . . . . . . . . .

Remission? □ nein □ ja Wenn ***ja***, am wievielten Krankenhaustag: . . . . . . . . . .

**7. Angaben zum stationären Aufenthalt**

Übernahme aus einer anderen Klinik? □ nein □ ja

Überweisung? □ nein □ ja Wenn ***ja***, von wem: □ Kinderarzt □ Hämatologie-Ambulanz

□ Hausarzt

Aufnahme auf Intensivstation? □ nein □ ja Wenn ***ja***, für wie viele Tage? . . . . . . . . . . Tage

Verlegung in eine andere Klinik? □ nein □ ja

**8. (Weiterer) ambulanter Verlauf**

Regelmäßige Blutbildkontrollen? □ nein □ ja Wenn ***ja***: □ mehrmals wöchentlich □ alle 2-4 Wochen

□ unbekannt □ wöchentlich □ seltener

Vorstellung nur bei Blutungszeichen? □ nein □ ja

□ unbekannt

Ambulante Interventionen: □ Transfusion □ Immunglobulingabe □ Steroidtherapie

□ anderes: . . . . . . . . . . . . . . . . . . . . . . . . . . . . . . . . . . . . . . . . . . . . . . . . . . . . . . . . . . . . . . . . . . . . . .

Niedrigster ambulanter Thrombozytenwert: . . . . . . . . . . . . . . . . . . /ml

**9. Angaben zum Krankenhaus**

Anzahl der ITP-Patienten/Jahr: □ 0-5 □ 6-10 □ >10

Intensivbetten: □ nein □ ja

Hämatologische Ambulanz: □ nein □ ja

Ambulante Betreuung durch die Klinik: □ nein □ ja

**10. Kommentare, Mitteilungen:**

**Vielen Dank für Ihre Mitarbeit!**
